# The Cage Case. Arts and Social Neuroscience

**DOI:** 10.3389/fsoc.2021.695991

**Published:** 2021-09-27

**Authors:** Amendola Alfonso, Jessica Camargo Molano

**Affiliations:** ^1^ Department of Political and Social Studies, University of Salerno, Rome, Italy; ^2^ Mind and Technologies in the Digital Society, Psychology Department, International Telematic University “UniNettuno”, Rome, Italy

**Keywords:** john cage, happening, art, music, theatre, neuroscience

## Abstract

A great story told by a musician is the basis of the best stage experimentation of the second half of the 20th century. The musician is John Cage, whose work synthesizes the entire system of arts within the extraordinary world of the avant-garde. This great story begins with the experimental artistic activities which were developed in the 1920s, consolidated in the thirties and continued through the post-war period up to the dawn of the fifties. Apart from the socio-historical cross-section Cage’s experimentation provides, it is also a pretext for reflecting on the artist’s work as well as the relationship between neuroscience and art. Important contributions to this topic come from the neuro-scientific-social research on new expressions “of creativity, imagination, genius” (Pecchinenda, 2018). This study is based on the assumption that Cage was the forerunner of neuronal experimentation that would be central to the experiments and research of many other artists. The theoretical reference model can be found in the research of the neuroscientist Kandel et al, whose work was the starting point for this investigation. Kandel grasps the definitive break between scientific logic and humanistic sensitivity in the methodological reductionism practiced by neuroscience and in the experiments of contemporary creativity. According to Kandel, both neuroscience and artistic experimentation have similar objectives and problems, and in some respects seem to develop similar methodological practices. Kandel identifies the use of memory, synthesis and knowledge of the world in authors such as Mondrian, de Kooning, Pollock, Rothko, Louis, Warhol as well as the New York school of which Cage was an important member. The relationship between art and neuroscience is synthetized in the avant-garde action of Cage and in all the artists who launched continuous attacks against traditional forms. The transition from figurative art to abstraction is “comparable” to the reductionist process that is used in the scientific field to explain complexity and phenomenology. The prolegomena of this discourse are anticipated by a previous work written by Kandel in 2012 and can also be found in other studies on the relationship between neuroscience and art, in particular in the reflections of the neurobiologist and father of neuroaesthetics, Semir Zeki. Zeki analyzed artists work as a practice perfectly comparable to the research carried out by neuroscientists. Cage, the focus of this investigation, carried out a sound-stage-vision experimentation affecting theatre, media and art which can be examined from at least two different perspectives. The first concerns the definitive subversion of “innate rules of perception” (Kandel) and the second deals with the relationship between art and neuroscience.

## Introduction

We often consider an artistic work as an experience belonging to the humanistic, social or cultural dimension, ignoring the biological component that contributes to creating it.

Art originates primarily in the functions and structures of the nervous system, which not only guarantees the entire apparatus of perceptive and executive possibilities but also pre-constitutes the linguistic architecture of the artistic work.

Among all artistic disciplines, music seems to be the one able to involve, simultaneously and deeply, the largest number of cortical and subcortical networks. This contingency has led to numerous studies on the “ability of musical practice to impose itself not only as an operational model to investigate the functioning of the brain in its complexity, but also as an enriching experience in terms of psychic activity, even independently of the cultural dimension”^1^.

This context is the starting point of the present research that analyzes the relationship between art and neuroscience. In particular, the analysis focuses on the work of John Cage, an artist who embodies the spirit of the avant-garde of the second half of the 20th century. Cage (1912–1992) is a central figure for any experience linked to contemporary experimentation. His work, which starts from the late surrealist rediscovery of Breton and Duchamp, ranges from *musique concr*è*te* and aleatory music to dance, from Zen to theatre and media.

According to Semir Zeki’s theories, artists act in the same way as neurologists who study the brain and its organization through their art. The function of the brain is to “grasp eternity in what is desperately transient”^2^, a function similar to that of art which has always tried to show the true nature of things beyond mere reproduction. This was accentuated by the advent of the camera: photography could perfectly reproduce objects and individuals, but painting could overcome the mere representation of the visible and show the invisible essence of things. Citing the French critic Jacques Rivière, Zeki points out that “the true purpose of painting is to represent objects as they are in reality, not as we see them. Painting always tends to give us their perceptible essence, their presence and that is the reason why its images do not resemble their ever-changing appearance”^3^.

Zeki’s definition compares two temporal concepts: eternity and transiency. Can art make what is transient eternal and is there a more transient form of art than *happening*? To answer these questions, the research focuses on John Cage who can be considered the father of *happening* for his performance at Black Mountain College in 1952. (De Marinis, 1987; Bonomo, Furghieri, 1998; Sylvester, 2012; Amendola, Frammenti d’immagine. Scene schermi video per una sociologia della sperimentazione, 2006; Cuomo, Distasio 2013; Fronzi 2014; Cage 2015)

Furthermore, Cage’s art is not an exclusively visual art; he staged musical experiments that stimulated the audience’s hearing and performances that implied movements. According to the concept that there is not one single aesthetic sense, Cage’s art can stimulate the different areas of the brain.

From neuroscientific studies, it has emerged that the inferior temporal cortex is the most involved part of the brain when an individual is observing or listening to a work of art, but it is not the only one. Art can involve the amygdala and, consequently, generate emotions. Therefore, art does not only make what is transient eternal, but also generates emotions.

Neurophysiologist Luciano Fadiga (2009); Fadiga (2019) associates the cerebral representations activated by art, in particular by music, with “a synaesthesia of a high order, which involves deep feelings, gives us the flavor of sensations and experiences, and makes the Spring by Vivaldi much more “spring” than the other three seasons of the same composition. This is due not only to the fact that the music sequence is in tune with the colors, scents, sounds of spring, but also to the fact that it evokes its movement, its destination”^4^.

Fadiga’s studies on music and on the areas of the brain activated at the moment of listening are in line with the work carried out by Cage, especially with regard to the analyses that the neurophysiologist carried out on the basis of Bukhard Maess studies. “By inserting unexpected harmonics Maess and co-workers created a sort of musical “syntactic” violation. Using magnetoencephalography (MEG), they studied the neuronal counterpart of hearing harmonic incongruity and they found an early right anterior negativity (ERAN) usually associated with harmonic violation”^5^.

These studies revealed the similarity between the network of the human brain involved in the elaboration of music and that of language processing. In particular, when an individual is subjected to unexpected musical chords, such as those that characterise Cage’s work, Broca’s and Wernicke’s areas, the superior temporal sulcus, Heschl’s gyrus, plana polaris and temporalis, and the anterior superior insular cortices are activated.

The object of the introduction is to explain the point of view from which Cage’s work will be analyzed in this paper which is organized as follows: *Materials and Methods* illustrates the sources and materials on which the research is based and the study methodologies used; *Results* analyzes John Cage’s artistic output, starting from the 1952 *happening* at Black Mountain College, in relation with some avant-garde artists of the second half of the 20th century; *Discussion* examines Cage’s work from a neuroscientific point of view.

## Materials and Methods

This essay can be defined a comparative research design, as the topic is analyzed from different points of view. In fact, the research is not limited to the artistic world, but it is also extended to the sociological and neuroscientific fields. The methodology used followed the phases as listed below:

### Reconstruction of the Object of Study Through Sources

The study is based on reports and analyses of Cage’s work. In particular, the sources consulted to study *happening* as an artistic phenomenon are the volume *Happening* by M. Kirby (1968) and “*Qualcosa deve succedere*” in *Nuovo teatro 1947–1970* by De Marinis (1987).

To understand the influence of Cage on the system of arts, important materials for the analysis are A. Amaducci (2014), *Videoarte. Storia, autori, linguaggi*, V. Valentini (2015), *Nuovo teatro made in Italy 1963–2013*, A. Amendola (2006), *Frammenti d’immagine. Scene schermi video per una sociologia della sperimentazione*, and A. Amendola (2012), *Videoculture. Storia, teorie ed esperienze artistiche dell’audiovisivo sperimentale.*

### Correlation Between the Object of Study and the Historical-Cultural Context

It was essential to set Cage’s work in the historical era in which the artist lived. Even in this case, the analysis was conducted through sources. As regards Oskar Schlemmer (1888–1943), Walter Adolph Gropius (1883–1969) and the other Bauhaus masters, the reference text is *Bauhaus* by Wingler, (1969), as well as the materials produced within the same school of art. For a reflection on the Bauhaus in contemporary society, the reference texts are A. Amendola and Camargo Molano, (2019), *Bauhaus, un*’*idea di sperimentazione*, and J. Camargo Molano (2021), *Bauhaus 2.0*.

### Insertion of the Object of Study in Schemes and Categories

This research aims to read John Cage’s work from a neuroscientific viewpoint. The innovative element of this study lies in the distinct perspective in reading Cage’s work.

In the neuroscientific field, the main references are the studies conducted by Eric Kandel and Semir Zeki. Analyzing John Cage’s artistic output, the research tries to adopt the same method used by Zeki in examining the receptive field (Mondrian and Malevič), kinetic art, neurology of abstract and figurative arts in *Inner Vision. An Exploration of Art and the Brain* (1999). The comparison with the most recent research in this sector was also very important. In particular, the studies of Stefan Koelsch (2009), Roger Beaty et al. (2014), Dennis Ramirez (2015), Davis Bashwiner et al. (2016), Kenneth Heilman (2016) and Wolfgang Mastnak (2020) were taken into consideration.

### Discussion on the Results

The neuroscientific reading of Cage’s work is studied and compared with recent theories on the relationship between music and human brain. This process leads to a reflection on what was analyzed.

## Results

It was the year 1952. John Cage’s sound experiments and aleatory music were already well known. He had founded his orchestra of percussion instruments only, created compositions for “prepared piano”, mixed extra-artistic objects in his musical production and revolutionized dance in collaboration with Merce Cunningham. Moreover, he had researched noise, silence and rejection of harmony and established his own orientation in the history of music towards the work of Henry Cowell and Edgard Varèse. In particular, he made it clear that music holds a continuous dialogue with all arts.

This section deals with the sensational *happening* that took place at Black Mountain College in North Caroline in the summer of 1952. It is a work rich in ideas, elements, procedures, proposals, language research, interference, quotations, games and fragmentations which characterize a large part of the subsequent media and stage experimentation. The stars M.C. Richards, Charles Olsen, David Tudor, Robert Rauschenberg and Merce Cunningham led by Cage, dressed in black, came on stage. This great event of the distant summer of 1952 was characterized by a free, albeit coherent explosion of linguistic forms that, starting from sound, ranged from painting to music, from cinema to improvisational gestural-instinctive theatre. Different languages and actions interacted with each other in fragmented scenes, creating a new dimension that had little to do with conventional theatre. The show, which was not staged by any director, had no guide and offered scenes full of irony and daring improvisation. It aimed at an experimental communication in which the language was of an auditory and visual nature. This is the aim of every *happening*: an expressive construction involving both the sense of hearing and that of seeing. Cage offered “a puzzle made of gestures, matters and noises”^6^. He invented a very innovative form of artistic expression. From then on, nothing was the same as before. In fact, the main and most significant experiences of *happening* show a continuous fragmentation of environments, cultural attitudes, linguistic constructs and trends. Recurring themes of this form of art are action, redundancy, repetition and difference. *Happening* uses a range of different spaces (art galleries, squares, beaches, warehouses), rejects any concert-theatre logic and creates irruptions, variations, new systems and forms. This triumph of fragmented creativity, which only an inattentive individual would identify as “chaos" without grasping the innovations of such creative experiences, is not an end in itself, or the result of a disorganized planning. *Happening* is characterized by a disrupted and not completely defined nature. It is a work in progress, “but this does not mean that happening has no structure”^7^. Its structure is based precisely on disruption, readaptation, contamination, new creativity.

It should be noted that some experiments had already appeared in the field of music research a few decades earlier. Among the abundant traces left by the historical avant-garde or even before, two names are noteworthy: Arnold Schönberg and Oskar Schlemmer. But it is also necessary to go back to Richard Wagner, whose vision includes the unification of all arts in *Gesamtkunstwerk*, a total art work. With *Der Ring des Nibelungen*, Wagner aimed *to bring the unconscious part of human nature to the level of consciousness* through the symbolism of theatrical actions, through an iconography that can be compared to a sort of interface design, where every sound motif reminds the spectators of an emotion, a concept, a character, an event. Schönberg tried to define *Gesamtkunstwerk* as a possible way to synthesize the totality of “musikdrama” in an abstract form of expression. It is no coincidence that *Die Gl*ü*ckliche Hand* was composed (1912–1913) in the same years in which Kandinsky painted his first pictures. Schönberg’s work, which expresses the moody visions of a man tormented by a continuous spiritual search, incorporates unprecedented stage techniques. Examples of these techniques include: a menacing choir of statues, mimic gestures illuminated by spotlights with a “ghost” effect and accompanied by mysterious chords, a rapid crescendo of lights together with changeable music structures through a series of fast, synchronized beats written on the score. Walter Gropius’s vision of theatre broke the scheme of the “spatial separation” between stage and auditorium with the aim of identifying an interactive medium that made the spectator participate in the play on the stage. Moholy-Nagy, continuing in the same direction and staging his mechanistic visions, created a synthesis between forms and movements, lights and sounds, colors and aromas. At the same time, Schlemmer planned performances based on the spatial dimensions of the human figure. *Circus* and variety entertainments combined with theatre and oriental puppets allowed him to create the new form of the theatre of illusion. Schlemmer’s thought focused not only on the human figure but also on the extension of the spatial dimensions of the stage. *Das Triadisches Ballet* embodies Schlemmer’s vision of *Gesamtkunstwerk*, a symbiosis of geometric elements influenced by machine technology and architecture, as well as by Kandiskian shapes and colors. The triad is made up of “t*he three parts of architectural composition and the fusion of dance, costumes and music*”^8^. The mechanistic choreography derives from the author’s passion for machines. And the actors? They are nothing more than dolls moving in a music box. On the stage, the geometric environment is balanced by joyful rhythms and bright colors and expands beyond the boundaries imposed by stage machinery. The multidisciplinary language is precise, coherent and abstract. As Schlemmer said, the stage, as an arena for progressive and transient actions, offers shapes and colors in motion. At first, they are showed in their primary aspect as separate and individually mobile entities, colored or uncolored, linear, flat or plastic and subsequently as transformable architectural structures, mobile and floating spaces. Such a kaleidoscope, that is both infinitely variable and tightly organized, would theoretically represent the absolute visual stage.

Besides these influences, it is worth mentioning the fact that, in the course of his experimentation, Cage also approached oriental philosophies becoming a Zen master. This knowledge of Zen emerges especially in Cage’s collaboration with the choreographer Merce Cunningham. “The collaboration of Merce Cunningham and John Cage brings together two art forms using minimalism and indeterminacy. Cunningham’s choreography explores the use of repetition and chance, Cunningham also used the I Ching (traditional Chinese text considered by Confucius to be the book of wisdom) that was passed along by Cage”^9^. The element that unites Cunningham’s choreographies and Cage’s works is chance: “His method of dance compositions in which the scenic designer, the composer, and the choreographer each worked independently of one another, knowing the climate of a dance but not its particulars”^10^. The two artists worked separately, Cage did not see Cunningham’s choreography and likewise Cunningham did not listen to Cage’s compositions. “By using “dancers” with no prior dance experience, Cunningham was able to teach them new movements without having preset movements in their body Performers now become audience members and audience members now become performers. Everyday people, not dancers, are asked for, hoped for. For the possibility of chance choreography, that new ideas not set movement would be expanded upon. The element of chance which has no real mood, meaning, and never a beginning or an ending thought was the biggest idea brought to concert dance. Nothing has to be or needs to be done for a reason. The music doesn’t drive the dance and the dance doesn’t persuade the music”^11^.

Dance plays a fundamental role in Cage’s work and it is also a point of reference in the neuroscientific framework of the artist’s activity. In particular, studies by Julia F. Christensen and Beatriz Calvo-Merino (2013) analysed which and how different areas of the brain are activated when a subject attends a dance performance.

^12^demonstrated that the perception of emotion expressed through bodily actions is related to activity in the superior temporal sulcus, recuneus and geniculate nucleus. Observing the emotional body movements of musicians performing a piece of music - shown in silence, without the music - also increases activity in the inferior parietal gyrus, insula and anterior cingulate cortex^13^. The identification of the emotion expressed in point views of human emotional movements is accompanied by increased activity in the EBA ^14^. The observation of whole-body emotional gestures is also related to increases in the activity in the EBA, as well as the ventral striatum ^15^ ^,^ ^16^.

The musical discourse, as a project of fragmentation of the scene and not only of the scene, held an absolute supremacy in the following decades. It contributed to developing a combination of formal and informal language, from which mass-media drew their terminology, fragmentation and reproduction as key elements of *happening*, and theatre as a fusion of genres and experiences. Thanks to Cage’s “creature”, *happening* materializedd a sound-stage-vision experimentation that affected theatre, media and art.

A reading, which is certainly outdated today, considered this form of art to be closely linked to certain forms of psychiatric therapy such as psychodrama. The “genre” was particularly influenced by the techniques used by Stanislavski in rehearsal to free actors from any inhibition and drive them to completely identify themselves with their characters. In the United States, a group of movie and theatre directors and actors (Elia Kazan, etc.) were attracted by these techniques, which they used to create successful films and plays through improvisation or free “expression”. It is probable that *happening* was influenced by the so-called *theatre of the absurd* and by some of Brecht’s experiments. It is clear that *happening* was only analyzed as a theatre product, ignoring the visual elements which were present in its fragmentation.

Today’s reader realizes that the 1952 *happening* and subsequent similar experiences are rich in socio-cultural elements, beginning from visual experimentation (action painting, new dada and subsequently pop-art) to the way of thinking about the function of theatre as a marked contamination of forms and styles.

The experimentation of *happening* can be better read through the magnifying lens of the historical avant-garde: from the editing of Futurism to the Dadaist improvisations of the Cabaret Voltaire in Zurich and to the surrealist exhibitions animated by Marcel Duchamp, from the structural technicality of the Bauhaus to the assemblages of Pop-art, and even *musique con*crète and serialism, avant-garde cinema, dance, poetry. It is therefore necessary to go back to the 1952 *happening* coordinated by Cage, which appears to be the most indicative moment of a new form of expression based on interrelation and fusion. Cage’s “concerted action” at Black Mountain College was not only an event that immediately became so famous that it is always mentioned, in more or less mythical terms, in any speech on the origins of the artistic neo-avant-garde in America, but also a synthetic anticipation of almost all the most important elements that characterize the proposals of the new theatre (American theatre and otherwise). Such elements are the participation of different artists (musicians, dancers, poets, painters), the type of actions, the way of organizing the space where these actions take place and the relationship between the artists on the stage and the public. Obviously, Cage continued carrying out his experiments throughout his career, influencing different sectors of the world avant-garde within and beyond the music field, including art, cinema, theatre, dance and many other spheres.

## Discussion

On the basis of his research, Eric Kandel asserts that “the reductionist approaches of scientists and artists are analogous: they both aim at the bases, at the nervous^17^ (Reductionism in Art and Brain Science, 2016).

Artists perform the same action of reductionism as scientists, but they do it with a different purpose: “while scientists use reductionism to solve complex problems, abstract artists exploit it to elicit a new perceptive and emotional response”^18^.

The emotion caused by observing or listening to a work of art is determined by the centers of affectivity and emotivity in the limbic system. According to Kandel’s studies, it is possible to state that every perceptive, emotional, mental or motor process is based “on distinct groups of specialized neuronal circuits located according to an ordered hierarchical arrangement in specific regions of the brain. The brain structures are anatomically and functionally linked to each other and therefore cannot be physically separated”^19^. It follows that art influences all the other categories of conscience and is influenced by them.

The analysis of Cage’s work fits into this theoretical framework. First of all, it is necessary to highlight that Kandel’s studies mainly focused on visual art, especially painting, a branch of art to which John Cage does not belong. Cage’s work is tipically musical, although the visual component of his works should not be absolutely neglected (just think of the 1952 *happening* at Black Mountain College).

Unlike the “visual brain” which consists of various areas, each of which is specializied in a specific function, the human brain does not have a single “music center”. Listening to music involves different networks distributed throughout the brain.

From the studies of Oliver Sacks (2011) it emerges that listening to music is not only an auditory, but also an emotional and motor experience. But the fundamental element in the analysis of music and consequently of Cage’s work, from a neuroscientific perspective is memory. “Much of what happens during the perception of music can also take place even when it is played in the mind. The imagination of music, even in relatively unmusical individuals, tends to be very faithful to the original: not only in melody and sentiment, but also in absolute pitch and time. At the basis of all that there is the extraordinary tenacity of music memory”^20^.

As stated by Stefan Koelsch^21^, listening to music activates mirror neurons: “mirror neurons are active during both perception and action, auditory working memory relies on sensorimotor codes that encode and maintain information, syntactic processing of music involves brain structures also involved in speech production, and the premotor cortex also serves a variety of cognitive tasks, such as working memory sequencing, and serial prediction.”

The cortical areas in charge of vision make use of two processes that Zeki calls *bottom-up* and *top-down*. The bottom-up process is based on higher-order mental and cognitive functions, first of all on memory, but also on attention, expectations and learned visual associations. The *top-down* process involves a creative task since the visual brain is required to integrate and complete the information that comes from the outside and is ambiguous or incomplete. For this reason, Kandel believes that the process activated by observing or listening to a work of art is a *top-down* process: the more ambiguous or essential the image is, the more active the observer’s imagination is.

Zeki comes to sthe same conclusion by carrying out a comparative analysis between abstract art and figurative art. “The works of art in conflict with the ordinary experience of the visual world - for example Magritte, De Chirico or Max Ernst - intensely involve the parts of the frontal lobe that are also activated by the paintings of the Fauves. In these works there is a conflict to be solved, that is the conflict between the present vision and the records of past experiences, and it seems that the frontal lobe is involved in this task”^22^.

Therefore, the analogy with Cage’s work is evident. John Cage offered his audience some new, unexpected sounds, different from what they had known until then. In observing a work of abstract art or listening to a work by Cage, the brain is required to perform that function that Kandel defines as a creative function. Observing shapes or listening to sounds that seem not to be part of his/her memory, an individual has to make an effort to decrypt, but above all, to complete the information he/she receives. Each individual will complete the information on the basis of his/her memory, reacting to observing or listening to the same work in a different way.

The creativity, which generates the work of art, also generates creativity in the public. It creates a circular link in which art and the public interact: art, as works originated by artistic gestures, makes the public, who enjoy them, artists, albeit within their own brain.

Neuroscience studies creativity through the underlying brain mechanisms (Heilman, 2016) and the dynamics of the brain network (Beaty et al., 2016).

Beaty asserts that creativity is based on the interactivity of different regions of the brain that define the default mode network (DMN), which is made up of the posterior cingulate cortex, the precuneus, the medial prefrontal cortex, the angular gyrus and the hippocampus.

Wolfgang Mastank^23^ thinks that DMN can be considered a form of inner intelligence, highly sensitive to music. For this reason it should be developed through an adequate musical education. This theory is shared by Bashwiner et al. (2016), who considers that “musically creative people have greater cortical surface area or volume in 1) regions associated with domain-specific higher-cognitive motor activity and sound processing 2) domain-general creative-ideation regions associated with the default mode network and 3) emotion-related regions These findings suggest that domain-specific musical expertise, default mode cognitive processing style, and intensity of emotional experience might all coordinate to motivate and facilitate the drive to create music”^24^.

From the analysis conducted, it emerges that John Cage’s work can be read from several points of view that, in some cases, intersect and influence each other.

Cage’s innovations are a cornerstone of the avant-garde of the 20th century. His experimentation influenced the history of music both of his period and the subsequent time. His innovative impetus can be also studied from a social point of view with the aim of understanding how Cage was able to revolutionize the world of art and his society.

Finally, the setting of Cage’s work in a neuroscientific context is grafted onto the broader scenario of contemporary research regarding the perception of music by the human brain. This analysis can be the starting point for revising the great masters of the avant-garde of the 20th century and reading their works in a different perspective.

## Data Availability

The original contributions presented in the study are included in the article/supplementary material, further inquiries can be directed to the corresponding author.

## Footnotes

1 Piricò, M. L., and Rigamonti, M. (2015). “La musica e le neuroscienze cognitive ed affettive: ricadute pedagogiche e scolastiche,” in Scuola Ticinese (Anno XLIV -Serie), IV. Bellinzona: DECS, 132, 55–59.

2 Zeki, S. (1999). Inner Vision. An Exploration of Art and the Brain. New York: Oxford University Press

3 Ibid.

4 Fadiga, L. (2019). Preface in Neuroscienze cognitive della musica. Il cervello musicale tra arte e scienza. Bologna: Zanichelli

5 Fadiga, L., Craighero, L., D’Ausilio, A. (2009). “Broca’s Area in Language, Action, and Music”, in The Neurosciences and Music III—Disorders and Plasticity: Ann. N.Y. Acad. Sci. 1169: 448–458 (2009). doi: 10.1111/j.1749-6632.2009.04582.x

6 Puppa, P. (1990). Teatro e spettacolo nel secondo Novecento. Roma-Bari: Laterza, p.268

7 Kirby, M. (1968). Happening. Bari: De Donato, p.16

8 Schlemmer, O. (1996). The theater of the Bauhaus. Baltimora: John Hopkins University Press

9 Morrison, J. M. (2005). “The Effects of Minimalism/Indeterminacy on the Merce Cunningham and John Cage Collaboration” in The Journal of Undergraduate Research (JUR) at Minnesota State University, volume 5, p.4

10 Kostelanetz, R. (1992) Merce Cunningham: Dancing in Space and Time. Atlanta: A Cappella Book, p.2

11 Morrison, J. M. (2005). “The Effects of Minimalism/Indeterminacy on the Merce Cunningham and John Cage Collaboration” in The Journal of Undergraduate Research (JUR) at Minnesota State University, volume 5, p.6

12 Van den Stock, J., Tamietto, M., Sorger, B., Pichon, S., Grézes, J., & deGelder, B. (2011). “Cortico-subcortical visual, somatosensory, and motoractivations for perceiving dynamic whole-body emotional expressionswith and without striate cortex (V1)” in Proceedings of the National Acad-emy of Sciences, USA, 108,16188–16193. doi:10.1073/pnas.1107214108

13 Petrini, K., Crabbe, F., Sheridan, C., & Pollick, F. E. (2011). “The music ofyour emotions: Neural substrates involved in detection of emotionalcorrespondence between auditory and visual music actions” in PLoS One, 6, e19165. doi:10.1371/journal.pone.0019165

14 Atkinson, A. P., Vuong, Q. C., & Smithson, H. E. (2012). “Modulation ofthe face- and body-selective visual regions by the motion and emotion ofpoint-light face and body stimuli” in NeuroImage, 59,1700–1712. doi:10.1016/j.neuroimage.2011.08.073

15 Peelen, M. V., & Downing, P. E. (2007). “The neural basis of visual bodyperception” in Nature Reviews Neuroscience, 8,636–648. doi:10.1038/nrn2195

16 Christensen, J. F., & Calvo-Merino, B. (2013). “Dance as a subject for empirical aesthetics” in Psychology of Aesthetics, Creativity, and the Arts, 7(1), 76–88. https://doi.org/10.1037/a0031827

17 Kandel, E.R. (2016). Reductionism in Art and Brain Science. New York: Columbia University Press

18 Ibid.

19 Ibid.

20 Sacks, O. (2011). Musicophilia: Tales of Music and the Brain. London: Pan Edition

21 Koelsch, S. (2009). “A Neuroscientific Perspective on Music Therapy” in The Neurosciences and Music III—Disorders and Plasticity: Ann. N.Y. Acad. Sci. 1169, p. 374–384

22 Zeki, S. (1999). Inner Vision. An Exploration of Art and the Brain. New York: Oxford University Press

23 Sangiorgio, A., Mastnak, W. (2020). Creative Interactions. Dynamic Processes in Group Music Activities. Munich: University of Music and Performing Arts Munich

24 Bashwiner, D.M., Wertz, C.J., Flores, R.A., & Jung, R.E. (2016). “Musical creativity “revealed” in brain structure: Interplay between motor, default mode, and limbic networks” in Scientific Reports, 6, 20482
